# Vicarious Hæmoptysis

**Published:** 1876-06

**Authors:** J. S. McCord

**Affiliations:** New Windsor, Ill.


### VICARIOUS HÆMOPTYSIS.

By J. S. McCORD, M.D., New Windsor, Ill.

(Read before the Military Tract Medical Society, at Galesburg, Ill., Jan. 11, 1876.)

I have to report a case of vicarious hæmoptysis, not using the term *vicarious*, perhaps, in its most commonly accepted sense—that is, one organ performing a function which properly belongs to another—but arguing that in this case the lungs take upon themselves the labor of a discharge which, in itself, was primarily morbid, viz a chronic abscess of cellulitis.

Mrs. N., aged forty, during a space of eighteen years, with the exception of three periods of pregnancy and nursing of usual duration, had almost every menstrual period followed by the formation of an abscess in the left ileo-uterine space. I think it is about a year and a half since I was called to see the patient, then suffering from one of those recurring attacks, somewhat more severe than previous ones, although she described them as all being "perfectly awful" until after occurrence of discharge, a period ranging from a few hours to three or four days. After thoroughly satisfying myself as to the nature of the case, I further verified my diagnosis by a physical examination, finding a large fluctuating tumor bulging into the vagina from the left side. I proposed to evacuate the pus. But this was objected to by the patient and her husband, on the ground that for eighteen years it had "broken itself," and when I argued the possibility of its discharge into the peritoneal cavity, the rectum or bladder, and so bringing about serious trouble, I was further assured that it had not only "broken," but "*broken in the right place.*" In due time the pus was discharged by an ulcerative process and the woman recovered as usual.

A few days later the patient came to my office desiring to know if anything could be done to prevent the constant return of her trouble. I advised a course of local treatment, with some constitutional remedies. But this advice, with something of her old spirit, she positively refused to accept. I then ordered the external application of tr. iod. over the seat of the abscess, as often as could be conveniently borne, together with potassic iodide and strychnia, thrice daily, internally.

I saw her from time to time, and ordered the treatment continued. To my great surprise, and at first to my delight, after a few months the abscess formation became less and less frequent, and at this date has not occurred for a period of nine months, although menstruation is still, as it has been, regular. The only trace of the abscess remaining, is a slight tenderness in the left iliac region. Now comes the point of interest in the case. Almost immediately upon the suppression of the abscess there commenced to occur the vicarious discharge of blood from the lungs in connection with or immediately following each menstrual flow. And to be strictly truthful, I should be forced to confess to my patient that I had, by interfering, actually induced a discharge from the wrong place.

